# Supplementary material for: Circulating Neoplastic-Immune Hybrid Cells Are Biomarkers of Occult Metastasis and Treatment Response in Pancreatic Cancer
Source: Cancers (Basel). 2024 Oct 29;16(21):3650. doi: 10.3390/cancers16213650 (PMC11545756; doi:10.3390/cancers16213650)
Supplement: Supplementary file 1 [file cancers-16-03650-s001.zip › cancers-3251692-supplementary.pdf]

cancers

Article

Circulating Neoplastic-Immune Hybrid Cells are Biomarkers of Occult Metastasis and Treatment Response in Pancreatic Cancer

Table S1. Antigens used in the phenotyping panel. Primary antibodies with fluor have been directly conjugated by the vendor. Those labeled "oligonucleotide" were used for oligonucleotide conjugation resulting in Ab-oligos.

Antibody	CAT #	Vendor	Clone	Dye	Dilution
Ki67	ab15580	Abcam	polyclonal	oligonucleotide	oligonucleotide
AKT	2920BF	Cell Signaling	40D4	oligonucleotide	oligonucleotide
pAKT	4060BF	CST	D9E	oligonucleotide	oligonucleotide
EGFR	ab174481	Abcam	EP38Y	oligonucleotide	oligonucleotide
CK	41-9003-82	Invitrogen	AE1/AE3	A570	1:50
CD45	41-9003-82	Life Technologies	HI30	A488	1:100
STING	NBP2-24683AF594	NOVUS	polyclonal	A647	1:200
